# Comprehensive Evaluation of Flavor in Charcoal and Electric-Roasted *Tamarix* Lamb by HS-SPME/GC-MS Combined with Electronic Tongue and Electronic Nose

**DOI:** 10.3390/foods10112676

**Published:** 2021-11-03

**Authors:** Yujun Xu, Dequan Zhang, Ruixia Chen, Xiaoyue Yang, Huan Liu, Zhenyu Wang, Teng Hui

**Affiliations:** 1 Institute of Food Science and Technology, Chinese Academy of Agricultural Sciences, Beijing 100193, China; xuyujun_96@163.com (Y.X.); zhangdequan@caas.cn (D.Z.); cchenruixia@163.com (R.C.); y550684149@163.com (X.Y.); liuhuan02@caas.cn (H.L.); wangzhenyu@caas.cn (Z.W.); 2 Key Laboratory of Agro-Products Processing, Ministry of Agriculture and Rural Affairs, Beijing 100193, China

**Keywords:** roasted *tamarix* lamb, charcoal roasting, electric roasting, taste, odor

## Abstract

To prevent the pollution generated during charcoal roasting of *tamarix* lamb, environmental-friendly electric is gradually applied in meat processing. The profile and formation of flavor in roasted *tamarix* lamb were evaluated using HS-SPME/GC-MS combined with E-nose/-tongue. Results indicated that charcoal-roasted *tamarix* lamb exhibited the higher taste of umami and sourness in E-tongue and had higher contents of alcohols, aldehydes, ketones, alkanes, and aromatics in E-nose, while the electric ones exhibited the higher taste of sweetness and bitterness and had higher contents of nitrogen oxides, terpenes, aromatics, and organic sulfur. Compared with charcoal, application of the electric significantly decreased the numbers of key volatile compounds with VIP > 1 (markers) and the contents of most markers.

## 1. Introduction

Roasted *tamarix* lamb, a characteristic meat product in northwest China, is very popular among consumers all over the country. Traditionally, the lamb cubes were skewered with *tamarix*, a special wood material, and roasted on the top of charcoal at 500–550 °C, then unique flavor were formed. *Tamarix* is considered as a potential substance to improve food quality due to its highly efficacious antioxidation and antibacterial properties [1]. *Tamarix* also contains abundant natural flavonoids compounds, which could inhibit the formation of heterocyclic amines in roasted lamb and conducive to meat safety [2]. It is a widely used as skewer to exert a good taste and a unique odor of roasted lamb in China, particularly in Xinjiang [3]. Recently, to prevent the pollution caused by traditional meat roasting, environmental-friendly electric roasting with maximum temperature of 500–550 °C was suggested to apply in meat processing. However, our latest findings indicated that the key volatile compounds with odor activity value greater than 1 of electric-roasted lamb decreased significantly, which reduced by 31% compared to charcoal roasting [4]. Among them, most of are aldehydes, which usually have a lower threshold and play a crucial role in the formation of flavor attributes in meat products. Therefore, it should not be ignored that the flavor attributes of the lamb could be compromised if charcoal roasting was replaced by electric ones, and comprehensive analysis should be carried out to better understand the flavor formation or flavor profile in charcoal and electric-roasted *tamarix* lamb.

The profile and formation of flavor in roasted lamb leg with charcoal, electric, microwave, and superheated steam roasted during processing have been analyzed comprehensively in our lab using headspace solid-phase microextraction combined with gas chromatography-mass spectrometry (HS-SPME/GC-MS) [4,5]. HS-SPME/GC-MS has been widely applied to evaluate the flavor profile of stir-fried pork slices [6], roasted beef [7], smoked chicken [8], and fried chicken nuggets [9]. However, there are some obvious limitations when evaluating the flavor of meat products using HS-SPME/GC-MS. Di Rosa et al. [10] reported that HS-SPME/GC-MS could not finely distinguish the flavor profile among different meat products. Sensor arrays of electronic nose (E-nose) and electronic tongue (E-tongue) are gradually used to distinguish food odor or taste due to their advantages of high sensitivity and excellent selectivity [11]. They are sensitive to odor or taste of meat or meat products. Slight changes in volatile or taste could cause significant differences in the responses of E-nose or E-tongue sensor [12,13], which make them effective tools to distinguish odor or taste [14]. Du et al. [15] reported that using HS-SPME/GC-MS combined with E-tongue and E-nose could clearly distinguish the difference in the flavor of bacon smoked with different woodchips, and the response data of E-nose/-tongue were highly correlated with the contents of aldehydes, alcohols, and ketones. Zhang et al. [8] also used HS-SPME/GC-MS combined with E-tongue and E-nose to investigate the flavor characteristics of chicken drumsticks smoked with sugar at different processing stages and found that there were correlations between sensors of E-nose and volatiles with odor activity value >1.

However, there are a few studies using E-tongue/-nose and HS-SPME/GC-MS to evaluate the flavor of roasted lamb. Therefore, the objectives of this study were (1) to comprehensively evaluate the profile and formation of flavor in charcoal and electric-roasted *tamarix* lamb using HS-SPME/GC-MS combined with E-tongue and E-nose; and (2) to reveal the key volatile compounds (markers) in charcoal and electric-roasted *tamarix* lamb at different processing stages using multivariate statistical methods combined with variable importance in the projection (VIP) procedure. We hope the results of this study would provide a reference for the profile and formation of flavor in traditional *tamarix* lamb roasted by charcoal and also for the application of other environmental-friendly thermal technologies for *tamarix* lamb in the future.

## 2. Materials and Methods

### 2.1. Chemicals and Reagents

2-methyl-3-heptanone (99%, GC-MS internal standard) was bought from Dr. Ehrenstorfer GmbH (Beijing, China). *n*-alkanes (C_7_–C_40_, 97%) standards were obtained from *o*2*si* Smart Solutions (Shanghai, China). Free fatty acid standards (98%) were obtained from Sigma-Aldrich Company (Shanghai, China).

### 2.2. Materials

A total of 30 male small fat-tail lambs (24.50 ± 1.08 kg, 7 months) from the same genetic and feeding system (same commercial diet and drylot feeding) were randomly selected. The lambs were slaughtered at a local abattoir in Xinjiang, China in accordance with the principles and guidelines established by the Animal Care and Use Committee of the Institute of Food Science and Technology, Chinese Academy of Agricultural Sciences described by Ding et al. [16]. A total of 30 *silversides* were trimmed off from the left side of the carcass at 24 h post-mortem (humidity 95%, temperature 4 °C) with ultimate pH of 5.62 ± 0.03. Then, the *silversides* were transported to a typical roast lamb restaurant in Xinjiang by cold-chain logistics within 1 h.

### 2.3. Roasted Tamarix Lamb Preparation

**Figure A1:**
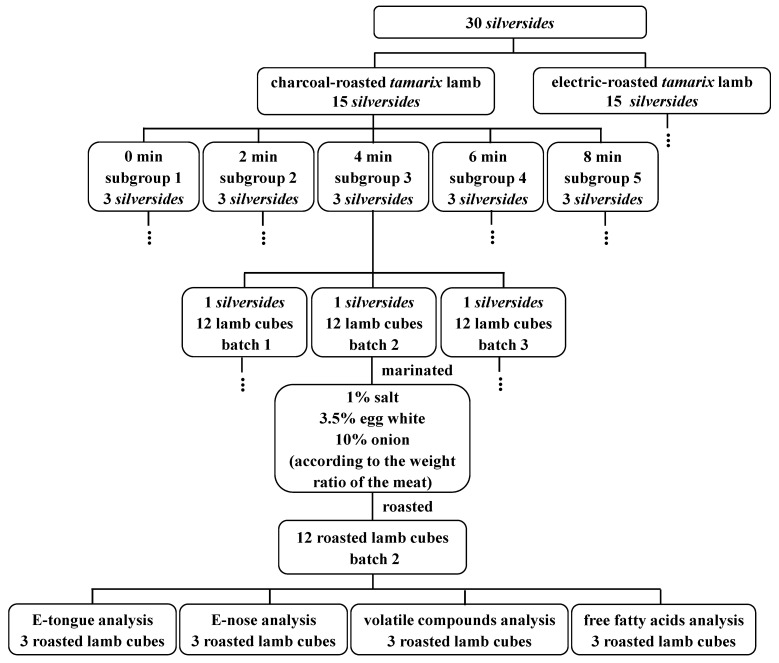
Flow chart showing the major roasted *tamarix* lamb preparation steps.

Thirty *silversides* were divided equally into two groups: charcoal-roasted *tamarix* lamb and electric-roasted *tamarix* lamb. Each group was further equally divided into five subgroups, including roasting for 0 min, 2 min, 4 min, 6 min, and 8 min (3 *silversides* in each subgroup). Each silverside was cut at 4 °C into 12 lamb cubes with uniform size of 3 × 3 × 1.5 cm, about 15 g for each cube. Then, each subgroup which had 36 lamb cubes was randomly divided into three batches (12 cubes in each batch). The 12 cubes from each batch represented one replicate sample. The samples were marinated with 1% salt, 3.5% egg white, and 10% onion (according to the weight ratio of the meat) at 25 °C for 2 h. After marinating, the cubes were skewered with *tamarix* stick, four cubes on each stick with 1 cm gap from each other. The major roasted *tamarix* lamb preparation steps can be seen in Figure A1 in Appendix A. All of the samples were roasted using a charcoal roasting equipment (SKJ-6082, LEPAIER, Shenzhen Lepaer Technology Co., Ltd. (Shenzhen, China), with digital temperature sensor) and an electric roasting oven (SJD-305-16, Xingguanyang Technology Co., Ltd., Wuxi, China) with the same temperature of 500–550 °C. The distance between the charcoal fire or electro-thermal tube and the roasted lamb was 5 cm. The roasted lambs were removed from the charcoal fire or oven at timepoints (0 min, 2 min, 4 min, 6 min, and 8 min), and then cooled at 25 °C for 2 min. At 8 min, the central temperature of the charcoal-roasted and electric-roasted lamb was 83.8 ± 2.36 °C and 80.7 ± 2.61 °C, respectively. After removing from the heat, all samples were wrapped immediately in nylon/polyethylene (9.3 mL O_2_/m^2^/24 h, 0 °C, 0.19 mm thick, Magic Seal^®^, Dongguan, China), put into liquid nitrogen and transported to our laboratory by cold-chain logistics. For the 12 roasted cubes in each batch, three cubes were used for E-tongue, E-nose, volatile compounds, and free fatty acids analysis, respectively.

### 2.4. E-Tongue Analysis

The E-tongue analysis of the roasted lamb was performed according to the method of Du et al. [15] with slight modifications. Taste attributes were analyzed using an electronic tongue (Astree, Alpha MOS, Toulouse, France) with 7 sensors and 1 reference electrode (Ag/AgCl). The AHS, CTS, NMS, ANS, and SCS sensors represent sourness, saltiness, umami, sweetness, and bitterness, respectively, and both the PKS and CPS sensors represent other complex tastes [17]. Briefly, the roasted *tamarix* lamb (40 g) was ground and then homogenized for 60 s with 200 mL distilled water at 40 °C, then the mixture was centrifuged at 8000 rpm for 15 min at 4 °C. The supernatant was used for E-tongue analysis after calibration and diagnosis of the sensors. The data acquisition sequence in E-tongue was conducted alternately with ultra-pure water and the final filtered liquid was used for the analysis, and the data acquisition time of each filter was 120 s. The detection results of E-tongue were converted to taste values using AlphaSoft v. 16 software (Alpha MOS, Toulouse, France).

### 2.5. E-Nose Analysis

The E-nose analysis of the roasted lamb was carried out according to Wang et al. [18] with slight modifications using a portable PEN3 electronic nose (Win Muster Airsense Analytics Inc., Schwerin, Germany). The PEN3 system consisted of 10 sensor probes, including W1C (aromatic compounds), W5S (nitrogen oxides), W3C (ammonia and aromatic compounds), W6S (hydrogen), W5C (alkanes and aromatics), W1S (methane, broad range of compounds), W1W (sulfur compounds, terpenes), W2S (alcohols, aldehydes and ketones), W2W (aromatics and organic sulfur compounds), and W3S (long-chain alkanes) [19,20]. Briefly, 1 g of ground roasted lamb was put into headspace of a 20 mL vial. The sensors absorbed the volatile gas of the sample through a hollow needle with a tube at 400 mL/min. The detection time was 60 s. After sampling, the clean air filtered by activated carbon was used to clean the sensor until the sensor signals returned to baseline. The response values of sensors were represented by the ratio of G to G0 (G, the conductance of the sensor after contacting the volatile components of the detected sample; G0, the conductance of the clean air).

### 2.6. Free Fatty Acid Analysis

The lipid of the roasted lamb was extracted as described by Liu et al. [21] with some modifications. Total of 40 mL of mixed chloroform-methanol solution (2:1, *v*/*v*) was mixed with 3 g of ground roasted lamb. The mixture was homogenized at 4500 r/min for 10 s, holding for 60 min, and the homogenate solution was filtered through Whatman No. 4 filter paper. The filtrate was washed with 8 mL of solution (0.5 g/L CaCl_2_ and 7.3 g/L NaCl) and centrifuged at 3000 r/min for 15 min. The total lipids were concentrated by a rotary evaporator at 45 °C. Free fatty acids were separated according to our previous method of Hui et al. [22]. The lipid extracts were fractionated by passing 20 mg of lipids dissolved in 5 mL of CHCl_3_: MeOH (2:1 *v*/*v*) through NH_2_-aminopropyl mini-columns. The mini-column first was washed with 2.0 mL of chloroform/isopropanol (2:1, *v*/*v*) to remove neutral lipids; then free fatty acids were obtained through eluting with 3.0 mL of 2% (*w*/*w*) acetic acid in diethyl ether. The free fatty acids and 10 µL of internal standard (heptadecanoic acid dissolved in hexane) were mixed with 1 mL 14% BF3/methanol (*m*/*m*). The mixture was methylated for 20 min at 50 °C in a water bath. After methylation, 4 mL of hexane was added into the mixture, shaken for 20 s and held for 1 h. Then methylated samples (1 μL) were analyzed using a gas chromatograph (GC-2010, Shimadzu, Kyoto, Japan) with a PEG-20 M capillary column (CP-Wax, 30 m × 0.32 mm × 0.25 μm). The oven temperature was maintained at 120 °C for 3 min, increased to 190 °C at 10 °C/min, and then increased to 230 °C at 3 °C/min, holding for 20 min. The detector and injection port temperature were maintained at 250 °C. The free fatty acids were identified by comparing the retention times of samples with those of standards and quantified with standard curve equations built with the standards of each fatty acid. The content of each fatty acid was expressed as mg/g dry matter.

### 2.7. HS-SPME/GC-MS for Volatile Compounds Analysis

The HS-SPME of the roasted lamb was carried out to extract volatile compounds according to Xu et al. [5]. Briefly, 2 g of ground roasted lamb was put into a 20 mL of headspace vial (Hamai Instrument Technology Co., Ltd., Ningbo, China), and 1.5 µL of 1.68 µg/μL 2-methyl-3-heptanone (internal standard) was added. The headspace vial was equilibrated at 50 °C for 20 min. Then the SPME fiber (DVB/PDMS extraction head, 65 μm, Supelco, Bellefonte, PA, USA) was exposed to the headspace of the vial at 50 °C for 40 min. Finally, the extraction head of the SPME fiber was transferred into a GC inlet and desorbed at 200 °C for 2 min.

The qualitative and quantitative of volatile compounds were carried out also according to Xu et al. [5] using a GC-MS system (QP2010 Shimadzu, Kyoto, Japan) with a DB-WAX (30 m × 0.25 mm × 0.25 μm). The initial oven temperature of GC was maintained at 40 °C for 3 min, then increased to 120 °C at a rate of 5 °C/min, finally increased to 200 °C at a rate of 10 °C/min, holding for 13 min. The temperature of the ion source was 200 °C. The MS was operated in full scan mode over a range of *m*/*z* 35–500. Volatile compounds were qualitatively identified by both the comparison of linear retention index (LRI) with authentic compounds and the mass spectrometry database (NIST) search. The contents of compounds were calculated by dividing the peak areas of the compounds by the peak area of the internal standard (2-methyl-3-heptanone) and multiplying this ratio by the initial concentration of the internal standard (expressed as ng/g).

### 2.8. Statistical Analysis

All data were performed through analysis of one-way ANOVA and Tukey’s test using SPSS version 19.0 software (IBM Corporation, Chicago, IL, USA) to determine the significant difference with *p* < 0.05. The principal component analysis (PCA) of E-tongue and E-nose was performed with Origin 2016 (Origin Lab, Hampton, MA, USA) to evaluate the changes of taste and odor of the lamb at different roasting times. Partial least squares regression (PLSR) model combined with variable importance in the projection (VIP) procedure was constructed to investigate the key volatile compounds in the charcoal and electric-roasted lamb. The roasting times were used as Y-variable, and the volatile compounds were used as X-variable, in which the compounds that significantly affected by roasting time or had greatest changes, as discriminant markers or volatile markers, were calculated with VIP value higher than 1 [23]. The PLSR model and VIP procedure were performed by the XLSTAT software (Version 2019, Microsoft, New York, NY, USA). All results were expressed as mean values ± standard error (SE), and all samples had three replicates.

## 3. Results and Discussion

### 3.1. E-Tongue Response in Roasted Tamarix Lamb

**Figure 1:**
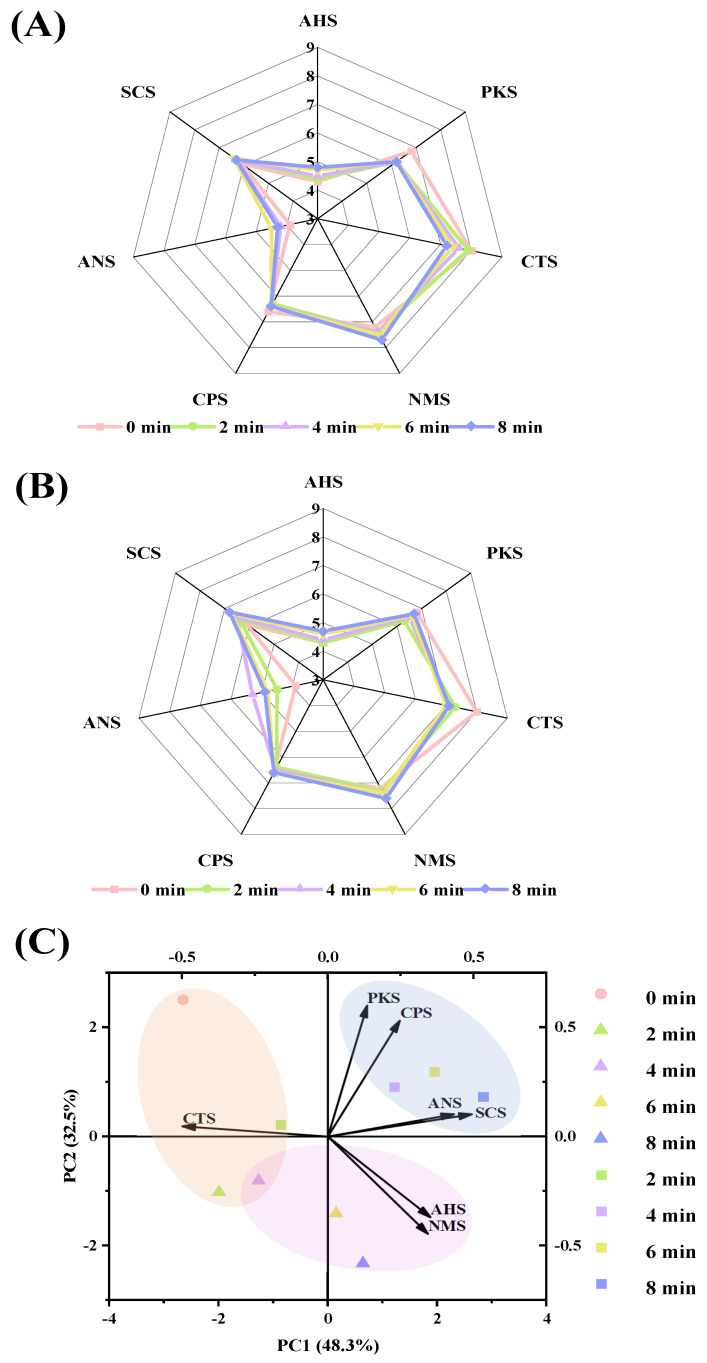
Radar chart of E-tongue in *tamarix* lamb roasted by charcoal (**A**) or electric (**B**) and their principal component biplots (**C**) (AHS, CTS, NMS, ANS, and SCS represented sourness, saltiness, umami, sweetness, and bitterness, respectively. PKS and CPS represented complex taste. Triangle or square represented *tamarix* lamb roasted by charcoal or electric, respectively).

The radar chart of E-tongue in roasted lamb is presented in Figure 1A,B. The NMS and CTS sensors had the greatest response values, indicating that the umami and saltiness were the predominant taste. Liu et al. [4] and Xu et al. [5] found that the most predominant taste characteristics of roasted lamb were the umami taste, except for the saltiness taste caused by the addition of salt. The taste profile of the product was mainly affected by free amino acids and 5′-nucleotides, among which the umami taste was formed from the synergistic effect of the flavor 5′-nucleotides and free amino acids [24]. Among them, glutamic acid was the most important umami contributor in roasted lamb. The principal component biplot of E-tongue is shown in Figure 1C. The contribution rates of the first and second principal component (PC) were 48.3% and 32.5%, respectively, accounting for 80.8% variance in total, which indicated that PC1 and PC2 fully explained the taste of roasted lamb [15]. The differences in taste between the charcoal and electric-roasted samples were mainly caused by PC2 and the projections of the two samples were located in the negative or positive axis of PC2, respectively, indicating that the taste attributes in these two samples were significantly different and could be completely separated by the E-tongue. The projections of the charcoal and the electric at different roasting times were clearly separated in the biplot, suggesting that with the roasting time, the taste of roasted lamb changed significantly. Within the initial 2 min of roasting time, both the charcoal and the electric were located in the negative axis of PC1, close to each other, indicating that there was no obvious difference in taste attribute between them, and they were located near to the CTS sensors, suggesting that roasted lamb exhibited salty taste. When roasting time extended from 4 min to 8 min, a clear separation of the projections was observed; the charcoal-roasted sample was located in the negative axis of PC2, near to NMS and AHS sensors, suggesting that they exhibited higher taste of umami and sourness in E-tongue. However, the electric-roasted samples were located in the positive axis of PC2, close to ANS and SCS sensors, indicating that they exhibited the higher taste of sweetness and bitterness in E-tongue, which were caused by sweet amino acids and bitter amino acids [4]. The results of E-tongue strongly suggested that compared with charcoal, application of the electric significantly changed the taste profiles of the roasted *tamarix* lamb.

### 3.2. E-Nose Response in Roasted Tamarix Lamb

**Figure 2:**
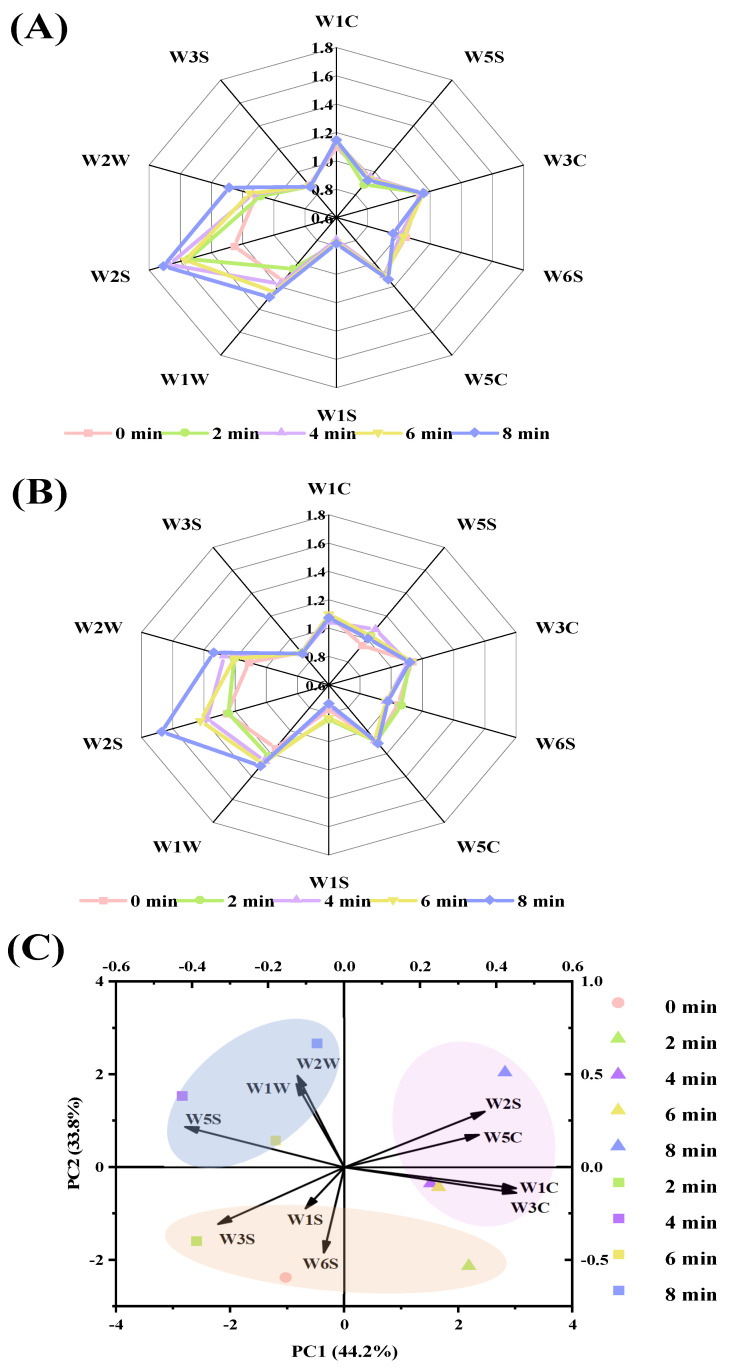
Radar chart of E-nose in *tamarix* lamb roasted by charcoal (**A**) or electric (**B**) and their principal component biplots (**C**) (W1C: aromatic compounds, W5S: nitrogen oxides, W3C: ammonia and aromatic compounds, W6S: hydrogen, W5C: alkanes and aromatics, W1S: methane, broad range of compounds, W1W: sulfur compounds, terpenes, W2S: alcohols, aldehydes and ketones, W2W: aromatics and organic sulfur compounds and W3S: long-chain alkanes. Triangle or square represented *tamarix* lamb roasted by charcoal or electric, respectively).

The radar chart of the E-nose in roasted lamb was presented in Figure 2A,B. The sensors, including W2S, W1W, W2W, W1C, W3C, and W5C, had stronger response values, which indicated that alcohols, aldehydes, ketones, alkanes, sulfides, and aromatic compounds were the predominant odor compounds. Liu et al. [25] also reported that the predominant odors of roasted lamb contained aldehydes, ketones, alcohols by flash GC E-nose and the results of GC-O-MS also showed that aldehydes and alcohols were the higher concentration chemicals in roasted lamb. Xiao et al. [26] and Liu et al. [4] also found that aldehydes and alcohols were the dominant compounds in roasted lamb by using HS-SPME/GC-MS. Du et al. [15] also found that smoked bacon might have higher contents of alcohols, aldehydes, ketones, and sulfides by E-nose. The principal component biplot of E-nose is shown in Figure 2C. The contribution rates of PC1 and PC2 were 44.2% and 33.8%, respectively, accounting for 78.0% variance in total, which fully explained the odor of roasted lamb [27]. The differences in odor between the charcoal and electric-roasted samples were mainly caused by PC1 and the projections of the two sample were located in the positive or negative axis of PC1, respectively, indicating that the odor attributes in these two samples were significantly different and could be completely separated by the E-nose. A clear separation of the projections was observed between charcoal and electric roasting from the beginning, suggesting that with extended roasting time, the odor of roasted lamb changed significantly. Within the initial 2 min of roasting, both the charcoal- and the electric-roasted samples were located in the negative axis of PC2, and they were near to W1S, W3S, and W6S sensors, which indicated that the samples had higher contents of hydrogen and methane. As roasting time extended from 4 min to 8 min, a clear separation of the projections was observed, and the charcoal-roasted sample was distributed in the positive axis of PC1, close to W1C, W3C, W2S, and W5C sensors, suggesting that they had higher contents of alcohols, aldehydes, ketones, alkanes, and aromatics. However, the electric-roasted sample was distributed in the negative axis of PC1, close to W5S, W1W, and W2W sensors, which suggested that they had higher contents of nitrogen oxides, terpenes, aromatics, and organic sulfur. The results of E-nose suggested that compared with charcoal, application of the electric significantly changed the odor profiles of roasted *tamarix* lamb.

### 3.3. Free Fatty Acids in Roasted Tamarix Lamb

Free fatty acids, as the precursor substance of volatile compounds, play a crucial role in the formation of flavor in meat products [28]. Seven kinds of free fatty acids were detected (Table 1), including three saturated fatty acids (SFA), two monounsaturated fatty acids (MUFA), and two polyunsaturated fatty acids (PUFA). Free fatty acids, including oleic acid (C_18:1_), palmitic acid (C_16:0_), linoleic acid (C_18:2_), and stearic acid (C_18:0_) were predominant, accounting for 89.83–90.99% and 89.68–91.29% of the total free fatty acids in the charcoal and the electric-roasted samples, respectively. At 2 min, SFA and MUFA increased significantly, and reached their maximum values. Oleic acid (C_18:1_) was the most abundant, followed by palmitic acid (C_16:0_), this obvious increase in the content of free fatty acid was most likely due to the lipolysis during roasting [21]. The contents of free fatty acids in the charcoal-roasted samples were higher than that of the electric-roasted ones. After 2 min of roasting, the contents of all free fatty acids decreased, their oxidation reactions at high temperature generated volatile compounds, leading to the decrease of their contents [29]. Two minute was a key control point for roasting, which could be used as a discriminant marker for a significant change of the precursors of volatile compounds in roasted lamb, and was also highly in line with the change trends of E-tongue and E-nose. At 8 min, the contents of the free fatty acids were higher in the charcoal-roasted samples than that of the electric-roasted ones. It was reported that linoleic acid and oleic acids could be pyrolyzed to produce hexanal [30], and octanal and nonanal [31], respectively. At 8 min, the lower contents of the linoleic acid and oleic acids after electric roasting indicated that electric roasting generated lower content of aldehydes compounds.

### 3.4. Volatile Compounds in Roasted Tamarix Lamb

#### 3.4.1. Profile of Volatile Compounds in Roasted *Tamarix* Lamb

**Figure 3:**
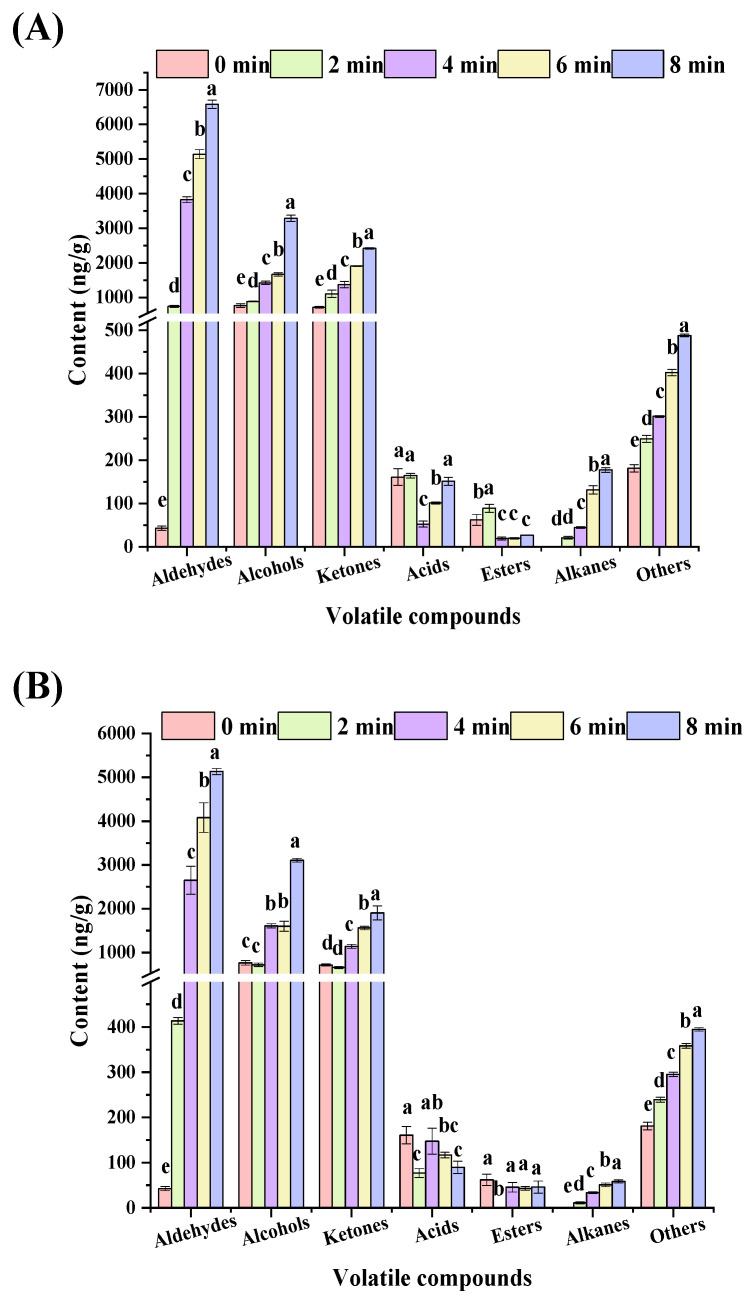
Profiles of volatile compounds in *tamarix* lamb roasted by charcoal (**A**) or electric (**B**). Different lowercase letters indicate that there is significant difference (*p* < 0.05).

A total of 40 and 34 volatile compounds were detected in charcoal and electric-roasted *tamarix* lamb, respectively (Table 2 and Table 3), including aldehydes, alcohols, ketones, esters, acids, alkanes, and other compounds, which was similar to the profiles of volatile compound in roasted lamb with our previous studies [4]. With extended roasting time, the total contents of volatile compounds were gradually increased (Figure 3A,B), with higher content in the charcoal-roasted samples than that of electric-roasted ones. The content of aldehydes was the highest, followed by alcohols, ketones. These three groups were the predominant, accounting for 79.00–94.08% and 79.00–94.51% of total volatile compounds in the charcoal and the electric-roasted samples, respectively, which was highly consistent with the highest response value of W2S, the sensor of aldehydes, alcohols, and ketones in the analysis of E-nose. Aldehydes are mainly formed by lipid oxidation, which usually make an important contribution to the overall flavor of meat products, due to their low threshold [32]. At 8 min, the types and total contents of aldehydes increased to their maximum values, higher in the charcoal-roasted samples with 15 types and 6579.13 ng/g than that of in electric-roasted samples with 12 types and 5134.61 ng/g, respectively (*p* < 0.05), the result was also highly consistent with the changes of linoleic acid and oleic acids, the precursor substance of aldehydes. Alcohols mainly come from the oxidation and degradation of lipids, with pleasant fruit and flower flavor [33], while ketones are also produced by oxidation of unsaturated fatty acids [34]. At 8 min, the total contents of alcohols and ketones were in the charcoal-roasted lamb with 3283.85 ng/g and 2415.85 ng/g, respectively, higher than that of in electric-roasted lamb with 3108.00 ng/g (*p* > 0.05) and 1903.95 ng/g (*p* < 0.05), respectively. The results of key profile of volatile compounds strongly suggested that compared with charcoal, application of the electric significantly reduced the lipid-types volatile compounds.

#### 3.4.2. Volatile Compound Markers in Roasted *Tamarix* Lamb

**Figure 4:**
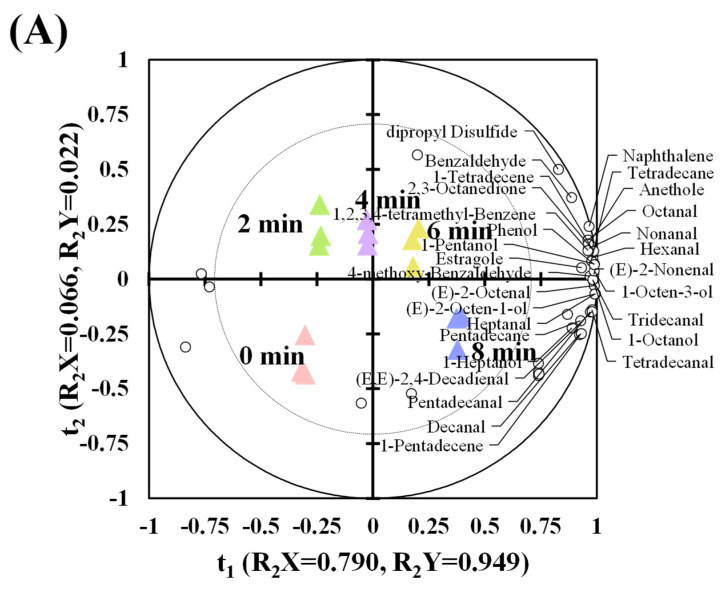

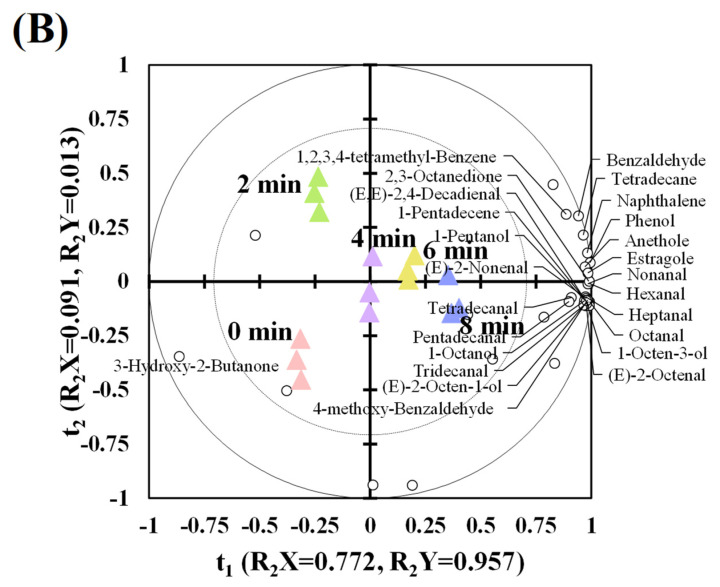
PLSR biplots showing the change of key volatile compounds in *tamarix* lamb roasted by charcoal (**A**) and electric (**B**) (triangle represented different roasting time, pink, green, purple, yellow, and blue represented 0 min, 2 min, 4 min, 6 min, and 8 min, respectively).

In order to clearly investigate the changes of key volatile compounds or volatile markers in charcoal and electric-roasted *tamarix* lamb, PLSR combined with VIP procedure was performed. The model parameters R^2^X and R^2^Y represent the interpretation rate of the established model to the X and Y matrices, and R^2^ describes how well the model fits. Q^2^ represents the predictive ability of the model. The biplot of PLSR shows that X variable (R^2^X = 0.856) was used to explain the Y variable (R^2^Y = 0.971) (Figure 4A) in the charcoal (*p* < 0.05, Q^2^ = 0.940), while X variable (R^2^X = 0.863) was also used to explain the Y variable (R^2^Y = 0.970) in the electric (*p* < 0.05, Q^2^ = 0.947) (Figure 4B), which demonstrated good fitting and predictability for the changes of volatile compounds. Charcoal-roasted lamb had a total of 29 volatile markers, including 13 aldehydes, 5 alcohols, 1 ketone, 4 alkanes and 6 other types, while electric-roasted lamb had 25 volatile markers, including 12 aldehydes, 4 alcohols, 2 ketones, 2 alkanes, and 5 other types (Table 4). Most of the markers were distributed in the positive direction of the t1, close to each other, and only 3-hydroxy-2-butanone was distributed in the negative direction of the t1. With extended roasting time, the projections of charcoal and electric-roasted lamb had similar migration trend in the biplot, at 0 min, their projections were located at the negative direction of t1 and t2. However, at 2 min, their projections quickly migrated to the positive of t2, suggesting there was a significant change in volatile makers in roasted lamb, which was highly in line with the changes of E-tongue, E-nose, and free fatty acids. As roasting time extended from 4 min to 8 min, their projections were gradually migrated from the negative of t1 and positive of t2 to positive of t1 and negative of t2, where most of the volatile markers had higher contents.

The 13 aldehydes markers in the charcoal-roasted lamb contained all of the 12 aldehydes markers that were detected in the electric-roasted lamb, and all the content of the aldehyde markers detected in electric-roasted lamb decreased compared with the charcoal-roasted lamb. Among all the aldehydes detected, hexanal was the highest, followed by nonanal, heptanal, and octanal. They were mainly formed by the oxidation degradation of unsaturated fatty acids [30]. It was reported that hexanal, nonanal, heptanal, and octanal presented grassy notes, floral notes, fat wax notes, and fruity notes, respectively [32]. (E)-2-nonenal and (E, E)-2,4-decadienal contributed to roasted meat odor, in addition, (E, E)-2,4-decadienal contributed to the odor of fat and oil [35]. The five alcohols markers in the charcoal-roasted lamb contained all of the four alcohols markers detected in the electric-roasted lamb, and electric roasting reduced the contents of most alcohol markers compared with charcoal roasting. In terms of alcohol compounds in roasted *tamarix* lamb, the content of 1-octene-3-ol was the highest, which comes from β-oxidation of fatty acids and exhibit mushroom odor [36]. 2,3-octadione and 3-hydroxy-2-butanone were the volatile markers in roasted *tamarix* lamb. 3-hydroxy-2-butanone and 2,3-octadione had sweet and buttery flavor [37,38]. Electric roasting reduced the content of 2,3-octadione, but increased the content of 3-hydroxy-2-butanone compared with the charcoal roasting. Alkanes come from homolysis of fatty acid alkoxy free radical and have higher odor threshold [39], therefore, they exerted less effect on the flavor of roasted *tamarix* lamb. These results suggested that compared with charcoal, application of the electric significantly decreased aldehydes and alcohols odors, eventually exhibiting more nitrogen oxides, terpenes, aromatics, and organic sulfur odor, which were detected by E-nose.

## 4. Conclusions

In order to investigate the formation and profiles of flavor in *tamarix* lamb roasted by charcoal and electric, HS-SPME/GC-MS combined with E-nose/-tongue was used to evaluate the profile and formation of flavor in charcoal and electric-roasted *tamarix* lamb at different roasting stages. The results indicated that the biplot projections of the charcoal and the electric-roasted lamb in E-tongue and E-nose at different roasting times were clearly separated. At 2 min, both the taste and odor of charcoal and electric-roasted lamb were close to each other. SFA and MUFA significantly increased, reaching to their maximum values. Oleic acid (C_18:1_) had the highest content, followed by palmitic acid (C_16:0_). As roasting time extended from 4 min to 8 min, the clear separations of the projections were observed in E-tongue and E-nose, and the contents of all free fatty acids decreased compared to 2 min, during the extended 4 min, the electric roasting reduced the numbers and the contents of volatile markers, especially aldehydes, alcohols, ketones. These results suggested that electric roasting could not fully replace charcoal to roast *tamarix* lamb without compromising the flavor of the final products.

## Figures and Tables

**Table 1:** Changes of free fatty acids in *tamarix* lamb roasted by charcoal or electric.

Free Fatty Acids (mg/g)	Roasted *Tamarix* Lamb	0 min	2 min	4 min	6 min	8 min
Myristic acid (C_14:0_)	charcoal	0.13 ± 0.02 ^c^	0.28 ± 0.03 ^Aa^	0.20 ± 0.02 ^Ab^	0.20 ± 0.01 ^Ab^	0.16 ± 0.02 ^Abc^
	electric	0.13 ± 0.02 ^c^	0.25 ± 0.02 ^Aa^	0.16 ± 0.02 ^Bbc^	0.18 ± 0.02 ^Ab^	0.13 ± 0.01 ^Bc^
Palmitic acid (C_16:0_)	charcoal	1.44 ± 0.12 ^d^	2.64 ± 0.03 ^Aa^	1.64 ± 0.18 ^Acd^	1.70 ± 0.11 ^Ac^	1.93 ± 0.09 ^Ab^
	electric	1.44 ± 0.12 ^b^	2.44 ± 0.12 ^Ba^	1.50 ± 0.32 ^Ab^	1.60 ± 0.21 ^Ab^	1.68 ± 0.08 ^Bb^
Palmitoleic acid (C_16:1_)	charcoal	0.13 ± 0.01 ^c^	0.30 ± 0.02 ^Aa^	0.21 ± 0.02 ^Ac^	0.21 ± 0.02 ^Ab^	0.16 ± 0.01 ^Ac^
	electric	0.13 ± 0.01 ^c^	0.27 ± 0.02 ^Aa^	0.17 ± 0.02 ^Ab^	0.18 ± 0.02 ^Ab^	0.13 ± 0.01 ^Bc^
Stearic acid (C_18:0_)	charcoal	0.99 ± 0.08 ^c^	1.34 ± 0.06 ^Aa^	0.86 ± 0.10 ^Ac^	0.94 ± 0.05 ^Ac^	1.19 ± 0.04 ^Ab^
	electric	0.99 ± 0.08 ^bc^	1.30 ± 0.05 ^Aa^	0.83 ± 0.17 ^Ac^	0.92 ± 0.14 ^Abc^	1.05 ± 0.05 ^Bb^
Oleic acid (C_18:1_)	charcoal	1.85 ± 0.26 ^d^	4.86 ± 0.19 ^Aa^	2.89 ± 0.18 ^Abc^	3.13 ± 0.17 ^Ab^	2.59 ± 0.25 ^Ac^
	electric	1.85 ± 0.26 ^c^	4.33 ± 0.24 ^Ba^	2.55 ± 0.13 ^Ab^	2.64 ± 0.30 ^Bb^	2.13 ± 0.14 ^Bbc^
Linoleic acid (C_18:2_)	charcoal	0.78 ± 0.13 ^c^	1.62 ± 0.11 ^Aa^	1.05 ± 0.10 ^Ab^	1.03 ± 0.05 ^Ab^	1.09 ± 0.05 ^Ab^
	electric	0.78 ± 0.13 ^b^	1.53 ± 0.16 ^Aa^	1.00 ± 0.10 ^Ab^	0.94 ± 0.10 ^Ab^	0.92 ± 0.02 ^Bb^
Arachidonic acid (C_20:4_)	charcoal	0.25 ± 0.06 ^b^	0.60 ± 0.11 ^Aa^	0.36 ± 0.04 ^Ab^	0.33 ± 0.03 ^Ab^	0.35 ± 0.02 ^Ab^
	electric	0.25 ± 0.06 ^b^	0.52 ± 0.08 ^Aa^	0.34 ± 0.03 ^Ab^	0.29 ± 0.03 ^Ab^	0.28 ± 0.02 ^Bb^
SFA	charcoal	2.74 ± 0.13 ^c^	4.61 ± 0.19 ^Aa^	2.97 ± 0.38 ^Ac^	3.05 ± 0.14 ^Ac^	3.58 ± 0.19 ^Ab^
	electric	2.74 ± 0.13 ^b^	4.30 ± 0.07 ^Ba^	2.75 ± 0.50 ^Ab^	2.95 ± 0.39 ^Ab^	3.15 ± 0.16 ^Bb^
MUFA	charcoal	1.84 ± 0.29 ^d^	5.29 ± 0.24 ^Aa^	3.18 ± 0.14 ^Abc^	3.45 ± 0.16 ^Ab^	2.73 ± 0.14 ^Ac^
	electric	1.84 ± 0.29 ^d^	4.63 ± 0.37 ^Ba^	2.75 ± 0.20 ^Bbc^	2.86 ± 0.25 ^Bb^	2.32 ± 0.15 ^Bc^
PUFA	charcoal	1.15 ± 0.26 ^c^	2.36 ± 0.35 ^Aa^	1.49 ± 0.19 ^Abc^	1.48 ± 0.01 ^Abc^	1.60 ± 0.16 ^Ab^
	electric	1.15 ± 0.26 ^b^	2.20 ± 0.22 ^Aa^	1.48 ± 0.19 ^Ab^	1.34 ± 0.16 ^Ab^	1.39 ± 0.01 ^Bb^

Note: Different lowercase letters (a–d) in the same row indicate significant differences between different roasting time (*p* < 0.05); different uppercase letters (A,B) in the same column indicate significant differences between different roasting methods at the same roasting time (*p* < 0.05). SFA: saturated fatty acids, MUFA: monounsaturated fatty acids, PUFA: polyunsaturated fatty acids.

**Table 2:** Volatile compounds in *tamarix* lamb roasted by charcoal (ng/g).

Compounds	LRI *^a^*		Identification *^d^*	0 min	2 min	4 min	6 min	8 min
	Literature *^b^*	Calculated *^c^*						
Aldehydes								
Hexanal	1074	1077	MS + LRI	ND	504.86 ± 19.38 ^d^	2523.37 ± 58.57 ^c^	3093.75 ± 177.33 ^b^	4181.43 ± 72.09 ^a^
Heptanal	1174	1178	MS + LRI	ND	ND	214.21 ± 11.94 ^c^	386.2 ± 7.97 ^b^	561.49 ± 36.34 ^a^
Octanal	1277	1284	MS + LRI	ND	46.61 ± 11.62 ^c^	206.50 ± 10.23 ^b^	316.89 ± 11.38 ^a^	325.40 ± 13.65 ^a^
Nonanal	1385	1384	MS + LRI	42.77 ± 4.78 ^d^	159.90 ± 11.98 ^c^	733.68 ± 22.78 ^b^	1105.67 ± 80.86 ^a^	1153.22 ± 20.39 ^a^
Decanal	1483	1493	MS + LRI	ND	ND	ND	13.72 ± 1.12 ^b^	24.09 ± 3.41 ^a^
Dodecanal	1710	1705	MS + LRI	ND	ND	ND	ND	11.93 ± 0.87 ^a^
Tridecanal	1824	1812	MS + LRI	ND	ND	16.22 ± 1.12 ^c^	26.64 ± 1.65 ^b^	34.92 ± 4.03 ^a^
Tetradecanal	1931	1920	MS + LRI	ND	ND	8.82 ± 0.15 ^c^	24.51 ± 4.89 ^b^	39.21 ± 1.90 ^a^
Pentadecanal	2042	2026	MS + LRI	ND	ND	6.17 ± 0.95 ^c^	16.63 ± 4.45 ^b^	26.77 ± 2.35 ^a^
Benzaldehyde	1508	1507	MS + LRI	ND	26.31 ± 1.03 ^c^	49.87 ± 3.93 ^b^	47.37 ± 2.24 ^b^	61.02 ± 1.65 ^a^
(E)-2-Octenal	1416	1419	MS + LRI	ND	ND	23.33 ± 0.23 ^c^	32.27 ± 2.05 ^b^	47.78 ± 1.15 ^a^
(E)-2-Nonenal	1535	1525	MS + LRI	ND	ND	24.87 ± 0.33 ^c^	40.80 ± 2.32 ^b^	49.77 ± 2.27 ^a^
(E,E)-2,4-Nonadienal	1686	1692	MS + LRI	ND	ND	ND	ND	11.97 ± 0.79 ^a^
(E,E)-2,4-Decadienal	1804	1801	MS + LRI	ND	ND	10.56 ± 0.31 ^c^	15.53 ± 1.71 ^b^	29.73 ± 0.59 ^a^
4-methoxy-Benzaldehyde	2014	2016	MS + LRI	ND	ND	8.64 ± 0.37 ^b^	19.30 ± 2.49 ^a^	20.40 ± 1.47 ^a^
Alcohols								
1-Pentanol	1252	1251	MS + LRI	59.32 ± 1.04 ^e^	80.37 ± 6.39 ^d^	154.04 ± 3.89 ^c^	225.08 ± 3.85 ^b^	251.58 ± 7.63 ^a^
1-Hexanol	1358	1353	MS + LRI	279.76 ± 29.76 ^c^	285.20 ± 14.65 ^c^	444.17 ± 37.59 ^b^	254.38 ± 27.68 ^c^	1616.85 ± 80.43 ^a^
1-Heptanol	1456	1455	MS + LRI	45.54 ± 6.21 ^c^	39.66 ± 8.38 ^c^	47.34 ± 3.00 ^c^	64.21 ± 4.93 ^b^	77.92 ± 9.22 ^a^
1-Octanol	1554	1557	MS + LRI	29.64 ± 2.98 ^d^	43.58 ± 4.68 ^c^	56.38 ± 1.20 ^c^	87.12 ± 3.16 ^b^	112.24 ± 11.34 ^a^
2,3-Butanediol	1570	1573	MS + LRI	20.37 ± 3.48 ^b^	23.85 ± 2.13 ^b^	20.15 ± 4.36 ^b^	42.43 ± 5.89 ^a^	48.68 ± 5.57 ^a^
1-Octen-3-ol	1451	1450	MS + LRI	290.96 ± 18.28 ^d^	353.60 ± 9.98 ^d^	606.96 ± 9.89 ^c^	847.64 ± 58.90 ^b^	973.18 ± 33.55 ^a^
(E)-2-Octen-1-ol	1617	1611	MS + LRI	36.86 ± 2.04 ^e^	55.78 ± 2.89 ^d^	91.56 ± 7.56 ^c^	144.92 ± 8.21 ^b^	203.41 ± 3.40 ^a^
Ketones								
2,3-Octanedione	1325	1325	MS + LRI	83.85 ± 11.63 ^e^	594.99 ± 94.84 ^d^	1312.26 ± 88.95 ^c^	1780.88 ± 11.36 ^b^	2337.90 ± 20.65 ^a^
3-Hydroxy-2-Butanone	1275	1278	MS + LRI	630.08 ± 32.87 ^a^	507.36 ± 80.31 ^b^	55.39 ± 5.51 ^c^	123.10 ± 19.81 ^c^	77.95 ± 1.82 ^c^
Acids								
Acetic acid	1441	1443	MS + LRI	22.12 ± 3.84 ^b^	25.51 ± 3.71 ^b^	ND	ND	43.29 ± 9.03 ^a^
Hexanoic acid	1849	1839	MS + LRI	117.86 ± 14.98 ^a^	101.74 ± 4.71 ^a^	52.20 ± 7.08 ^b^	101.00 ± 2.30 ^a^	107.90 ± 5.63 ^a^
Nonanoic acid	2169	2162	MS + LRI	20.75 ± 8.04 ^b^	37.03 ± 2.91 ^a^	ND	ND	ND
Esters								
*n*-Caproic acid vinyl ester	-	1613	MS	62.07 ± 12.64 ^b^	88.57 ± 9.13 ^a^	18.70 ± 3.42 ^c^	19.53 ± 1.64 ^c^	26.31 ± 0.19 ^c^
Hydrocarbons								
Tetradecane	-	1400	MS	ND	8.60 ± 1.99 ^c^	19.46 ± 1.30 ^b^	47.04 ± 1.36 ^a^	50.82 ± 3.88 ^a^
Pentadecane	-	1500	MS	ND	ND	ND	26.49 ± 4.34 ^b^	36.38 ± 1.02 ^a^
1-Tetradecene	1446	1444	MS + LRI	ND	12.10 ± 3.13 ^c^	24.85 ± 2.96 ^b^	31.62 ± 4.75 ^b^	45.39 ± 1.05 ^a^
1-Pentadecene	1545	1545	MS + LRI	ND	ND	ND	26.12 ± 0.88 ^b^	44.56 ± 0.87 ^a^
Others								
Phenol	1992	1994	MS + LRI	ND	6.51 ± 0.52 ^d^	11.03 ± 0.26 ^c^	22.32 ± 0.60 ^b^	28.60 ± 0.56 ^a^
Anethole	1818	1817	MS + LRI	25.74 ± 0.11 ^d^	44.15 ± 4.97 ^c^	69.57 ± 1.42 ^b^	117.40 ± 1.90 ^a^	116.75 ± 6.63 ^a^
Estragole	1624	1601	MS + LRI	19.45 ± 3.02 ^e^	25.45 ± 3.55 ^d^	34.06 ± 2.57 ^c^	47.77 ± 2.48 ^b^	57.76 ± 0.82 ^a^
Naphthalene	1722	1723	MS + LRI	ND	7.53 ± 0.21 ^d^	14.33 ± 0.51 ^c^	22.56 ± 1.43 ^b^	24.56 ± 0.44 ^a^
2-pentyl-Furan	1230	1216	MS + LRI	ND	ND	ND	ND	78.12 ± 0.14 ^a^
dipropyl Disulfide	1365	1301	MS + LRI	12.48 ± 2.45 ^c^	30.63 ± 4.64 ^b^	37.39 ± 2.26 ^ab^	40.93 ± 6.02 ^a^	43.34 ± 1.90 ^a^
methoxy-phenyl-Oxime	-	1773	MS	93.69 ± 4.25 ^a^	102.13 ± 6.65 ^a^	94.61 ± 2.71 ^a^	103.84 ± 1.87 ^a^	97.48 ± 3.88 ^a^
1,2,3,4-tetramethyl-Benzene	1430	1424	MS + LRI	29.64 ± 3.53 ^d^	36.18 ± 2.89 ^c^	43.39 ± 3.16 ^b^	54.66 ± 1.60 ^a^	57.61 ± 2.46 ^a^

Note: Different lowercase letters in the same row indicate that there is significant difference (*p* < 0.05). ^*a*^ Linear retention index. *^b^* Reported data. *^c^* Calculated data based on *n*-alkanes (C_7_–C_40_). *^d^* Means of identification: MS, mass spectrum comparison using NIST libraries; LRI, linear retention index compared with literature values. ND: volatile compounds not detected. “-”: not reported in the literature.

**Table 3:** Volatile compounds in *tamarix* lamb roasted by electric (ng/g).

Compounds	LRI *^a^*		Identification *^d^*	0 min	2 min	4 min	6 min	8 min
	Literature *^b^*	Calculated *^c^*						
Aldehydes								
Hexanal	1074	1077	MS + LRI	ND	269.19 ± 13.98 ^d^	1679.38 ± 234.34 ^c^	2502.59 ± 299.27 ^b^	3162.92 ± 73.81 ^a^
Heptanal	1174	1178	MS + LRI	ND	ND	287.38 ± 17.70 ^c^	383.43 ± 53.52 ^b^	485.33 ± 36.53 ^a^
Octanal	1277	1284	MS + LRI	ND	ND	137.71 ± 10.66 ^c^	231.66 ± 19.25 ^b^	322.47 ± 11.91 ^a^
Nonanal	1385	1384	MS + LRI	42.77 ± 4.78 ^d^	122.51 ± 7.35 ^d^	453.60 ± 91.57 ^c^	817.94 ± 50.57 ^b^	963.53 ± 66.37 ^a^
Tridecanal	1824	1812	MS + LRI	ND	ND	9.65 ± 1.08 ^c^	15.52 ± 1.14 ^b^	19.39 ± 1.24 ^a^
Tetradecanal	1931	1920	MS + LRI	ND	ND	ND	9.84 ± 0.63 ^b^	15.46 ± 1.25 ^a^
Pentadecanal	2042	2026	MS + LRI	ND	ND	ND	8.84 ± 0.91 ^b^	17.57 ± 2.16 ^a^
Benzaldehyde	1508	1507	MS + LRI	ND	21.60 ± 1.09 ^d^	30.38 ± 1.22 ^c^	39.88 ± 1.15 ^b^	48.12 ± 3.09 ^a^
(E)-2-Octenal	1416	1419	MS + LRI	ND	ND	17.61 ± 1.70 ^b^	22.33 ± 2.27 ^b^	33.96 ± 5.34 ^a^
(E)-2-Nonenal	1535	1525	MS + LRI	ND	ND	21.97 ± 2.98 ^c^	29.40 ± 4.30 ^b^	37.85 ± 4.24 ^a^
(E,E)-2,4-Decadienal	1804	1801	MS + LRI	ND	ND	5.39 ± 0.37 ^c^	9.84 ± 0.81 ^b^	15.45 ± 0.78 ^a^
4-methoxy-Benzaldehyde	2014	2016	MS + LRI	ND	ND	6.94 ± 0.37 ^c^	10.22 ± 0.61 ^b^	12.55 ± 0.63 ^a^
Alcohols								
1-Pentanol	1252	1251	MS + LRI	59.32 ± 1.04 ^c^	61.07 ± 0.95 ^c^	170.54 ± 20.56 ^b^	204.90 ± 16.17 ^b^	323.82 ± 38.11 ^a^
1-Hexanol	1358	1353	MS + LRI	279.76 ± 29.76 ^c^	275.08 ± 16.25 ^c^	603.41 ± 24.02 ^b^	359.60 ± 48.40 ^c^	1560.68 ± 66.70 ^a^
1-Heptanol	1456	1455	MS + LRI	45.54 ± 6.21 ^bc^	37.87 ± 7.06 ^c^	92.97 ± 25.34 ^a^	62.05 ± 10.15 ^bc^	65.05 ± 8.93 ^b^
1-Octanol	1554	1557	MS + LRI	29.64 ± 2.98 ^c^	32.02 ± 4.92 ^c^	69.48 ± 2.39 ^b^	71.70 ± 8.00 ^b^	96.38 ± 6.37 ^a^
2,3-Butanediol	1570	1573	MS + LRI	20.37 ± 3.48 ^b^	6.97 ± 0.85 ^c^	19.36 ± 7.26 ^b^	29.91 ± 4.75 ^b^	46.70 ± 2.74 ^a^
1-Octen-3-ol	1451	1450	MS + LRI	290.96 ± 18.28 ^d^	266.46 ± 28.95 ^d^	572.64 ± 43.02 ^c^	767.44 ± 40.58 ^b^	870.85 ± 28.10 ^a^
(E)-2-Octen-1-ol	1617	1611	MS + LRI	36.86 ± 2.04 ^d^	37.07 ± 1.79 ^d^	79.21 ± 7.81 ^c^	103.55 ± 7.15 ^b^	144.51 ± 27.38 ^a^
Ketones								
2,3-Octanedione	1325	1325	MS + LRI	83.85 ± 11.63 ^e^	296.36 ± 24.19 ^d^	972.63 ± 40.95 ^c^	1451.35 ± 40.10 ^b^	1791.11 ± 180.50 ^a^
3-Hydroxy-2-Butanone	1275	1278	MS + LRI	630.08 ± 32.87 ^a^	361.35 ± 47.65 ^b^	166.85 ± 22.42 ^c^	107.16 ± 21.54 ^c^	112.84 ± 29.54 ^c^
Acids								
Acetic acid	1441	1443	MS + LRI	22.12 ± 3.84 ^b^	ND	33.53 ± 5.48 ^a^	ND	ND
Hexanoic acid	1849	1839	MS + LRI	117.86 ± 14.98 ^a^	53.10 ± 12.05 ^b^	84.48 ± 15.45 ^b^	92.06 ± 2.21 ^b^	89.89 ± 13.63 ^c^
Nonanoic acid	2169	2162	MS + LRI	20.75 ± 8.04 ^a^	24.12 ± 6.77 ^a^	29.53 ± 10.48 ^a^	24.81 ± 4.35 ^a^	ND
Esters								
*n*-Caproic acid vinyl ester	-	1613	MS	62.07 ± 12.64 ^a^	ND	45.81 ± 10.45 ^a^	43.06 ± 4.14 ^a^	46.21 ± 13.53 ^a^
Hydrocarbons								
Tetradecane	-	1400	MS	ND	11.28 ± 2.05 ^d^	19.09 ± 0.78 ^c^	27.98 ± 1.34 ^b^	31.52 ± 1.40 ^a^
1-Pentadecene	1545	1545	MS + LRI	ND	ND	14.84 ± 2.44 ^b^	22.95 ± 3.11 ^a^	27.02 ± 2.00 ^a^
Others								
Phenol	1992	1994	MS + LRI	ND	4.33 ± 0.03 ^d^	9.58 ± 1.05 ^c^	15.88 ± 0.46 ^b^	21.18 ± 0.58 ^a^
Anethole	1818	1817	MS + LRI	25.74 ± 0.11 ^d^	36.35 ± 0.73 ^c^	57.68 ± 5.32 ^b^	85.51 ± 5.92 ^a^	87.70 ± 0.95 ^a^
Estragole	1624	1601	MS + LRI	19.45 ± 3.02 ^c^	21.94 ± 3.80 ^c^	30.55 ± 1.93 ^b^	40.45 ± 2.57 ^a^	41.37 ± 4.58 ^a^
Naphthalene	1722	1723	MS + LRI	ND	4.92 ± 0.72 ^d^	7.78 ± 0.74 ^c^	13.30 ± 0.63 ^b^	17.92 ± 0.38 ^a^
dipropyl Disulfide	1365	1301	MS + LRI	12.48 ± 2.45 ^c^	36.62 ± 3.32 ^b^	42.47 ± 3.29 ^ab^	47.27 ± 3.38 ^a^	50.99 ± 6.55 ^a^
methoxy-phenyl-Oxime	-	1773	MS	93.69 ± 4.25 ^a^	90.74 ± 5.68 ^a^	105.31 ± 5.91 ^a^	99.38 ± 8.88 ^a^	103.02 ± 1.51 ^a^
1,2,3,4-tetramethyl-Benzene	1430	1424	MS + LRI	29.64 ± 3.53 ^c^	40.77 ± 3.02 ^b^	38.41 ± 2.09 ^b^	49.11 ± 2.55 ^a^	55.94 ± 5.05 ^a^

Note: Different lowercase letters in the same row indicate that there is significant difference (*p* < 0.05). *^a^* Linear retention index. *^b^* Reported data. *^c^* Calculated data based on *n*-alkanes (C_7_–C_40_). *^d^* Means of identification: MS, mass spectrum comparison using NIST libraries; LRI, linear retention index compared with literature values. ND: volatile compounds not detected. “-”: not reported in the literature.

**Table 4:** The volatile compounds with VIP >1 (markers) of *tamarix* lamb roasted by charcoal or electric.

Variable	VIP	
	Charcoal	Electric
Hexanal	1.114	1.121
Heptanal	1.107	1.108
Octanal	1.088	1.116
Nonanal	1.085	1.106
Decanal	1.009	—
Tridecanal	1.107	1.112
Tetradecanal	1.088	1.030
Pentadecanal	1.069	1.019
Benzaldehyde	1.068	1.114
(E)-2-Octenal	1.104	1.102
(E)-2-Nonenal	1.105	1.106
(E, E)-2,4-Decadienal	1.078	1.105
4-methoxy-Benzaldehyde	1.076	1.097
1-Pentanol	1.115	1.110
1-Heptanol	1.008	—
1-Octanol	1.127	1.108
1-Octen-3-ol	1.108	1.099
(E)-2-Octen-1-ol	1.111	1.096
2,3-Octanedione	1.131	1.117
3-Hydroxy-2-Butanone	—	1.018
Tetradecane	1.104	1.135
Pentadecane	1.016	—
1-Tetradecene	1.134	—
1-Pentadecene	1.012	1.087
Phenol	1.123	1.144
Anethole	1.106	1.108
Estragole	1.005	1.122
Naphthalene	1.120	1.139
dipropyl Disulfide	1.032	—
1,2,3,4-tetramethyl-Benzene	1.104	1.075

Variable importance in the projection, VIP; “—” means VIP < 1.

## Data Availability

The data presented in this study are available on request from the corresponding author.
